# Reproducibility of slice-interleaved myocardial T_2_ mapping sequences

**DOI:** 10.1186/1532-429X-18-S1-P54

**Published:** 2016-01-27

**Authors:** Steven Bellm, Tamer Basha, Long Ngo, Sophie Berg, Kraig V Kissinger, Beth Goddu, Warren J Manning, Reza Nezafat

**Affiliations:** 1 Medicine, Beth Israel Deaconess Medical Center, Harvard Medical School, Boston, MA USA; 2 Radiology, Beth Israel Deaconess Medical Center, Harvard Medical School, Boston, MA USA

## Background

Myocardial T_2_ mapping sequence allows quantitative assessment of myocardial edema and inflammation. Commonly, a series of T_2_ weighted images with steady-state free-precession (SSFP) are acquired after T_2_ magnetization preparation (T_2_Prep) with different echo times. Conventionally, a single slice per breath-hold is acquired to image one single slice. Because inflammation/edema is often regional, multiple breath-holds are needed to cover the entire ventricle. The slice-interleaved T_2_ mapping sequence was recently proposed to image multiple slices in a single scan by using a slice-selective T_2_Prep. While accuracy of this sequence to quantify T_2_ was previously studied, the measurement reproducibility is not known. Therefore, we sought to investigate the reproducibility of myocardial T_2_ mapping using the slice-interleaved T_2_ mapping sequence.

## Methods

Eleven healthy subjects (age: 33 ± 16 years, 6 males) were imaged on 2 different days with the same scan protocol using a 1.5T MRI scanner (Philips Achieva). On each day, slice-interleaved T_2_ sequence was repeated twice. Subsequently, subjects were removed from the scanner and repositioned, followed by another 2 repetitions of the same scan. The following imaging parameters were used: In-plane resolution = 2.1 × 2.1 mm^2^, slice thickness = 8 mm, slice gap = 4 mm, Field of View = 320 × 320 mm^2^, TR/TE/α = 2.8 msec. / 1.38 msec. /55°, SENSE-rate = 2.3, and acquisition window = 191 ms, bandwidth = 1879.7 Hz/pixel. Motion correction was performed between different images. T_2_ maps were calculated using a 3-parameter fit model. The epicardial and endocardial contours in the left ventricle were manually drawn in 5 short axis-slices to calculate global and slice-based myocardial T_2_ values. Coefficient of variation (CV) analysis for each slice was generated to assess the variability. Bland-Altman plots were used to test for significant differences between repetitions, sessions and days.

## Results

**Figure 1:**
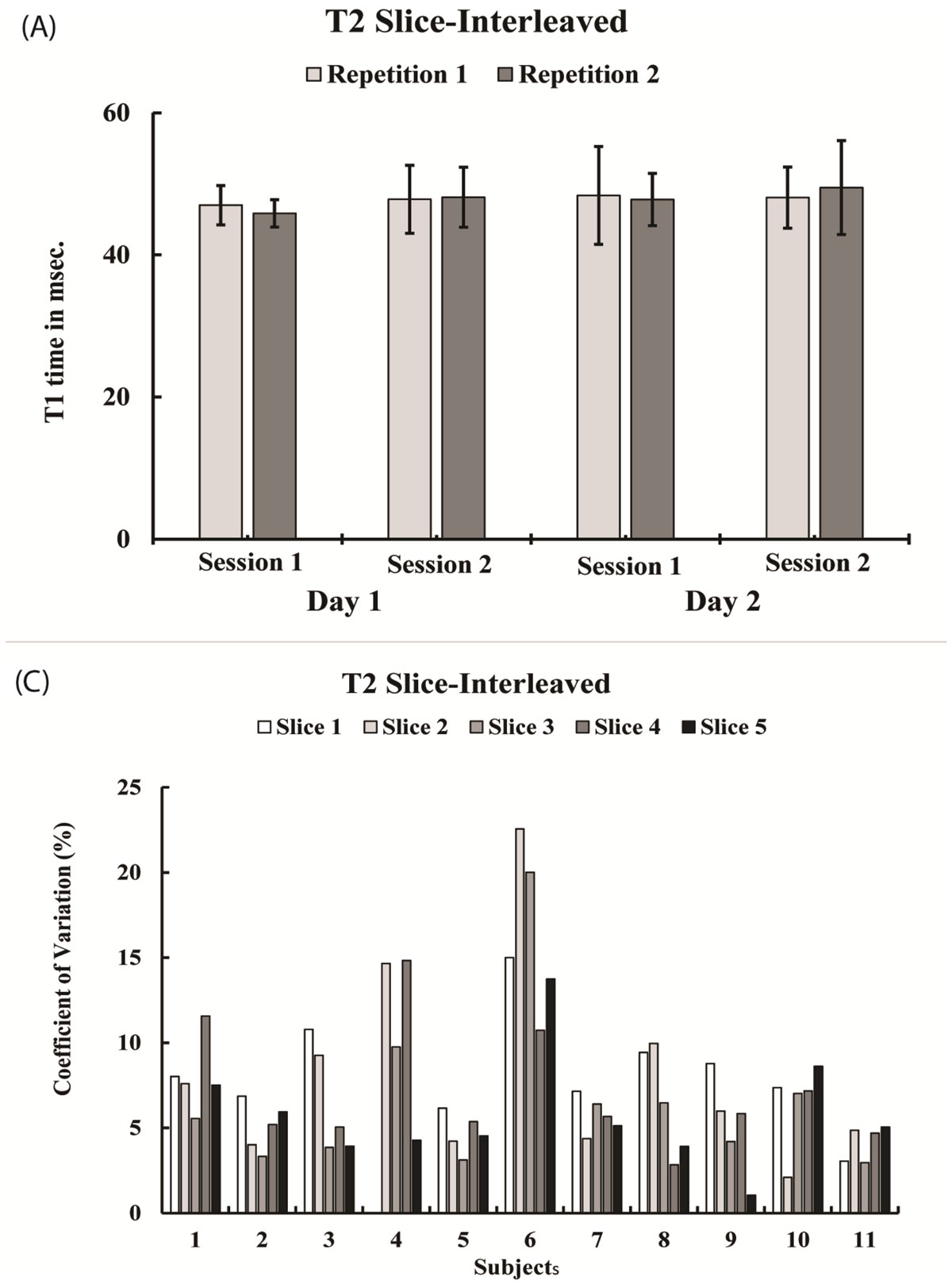
**Mean T2 estimates per repetition, session and day (A) and coefficients of variation for different slices and subjects for slice-interleaved T2 sequence (B)**.

**Figure 2:**
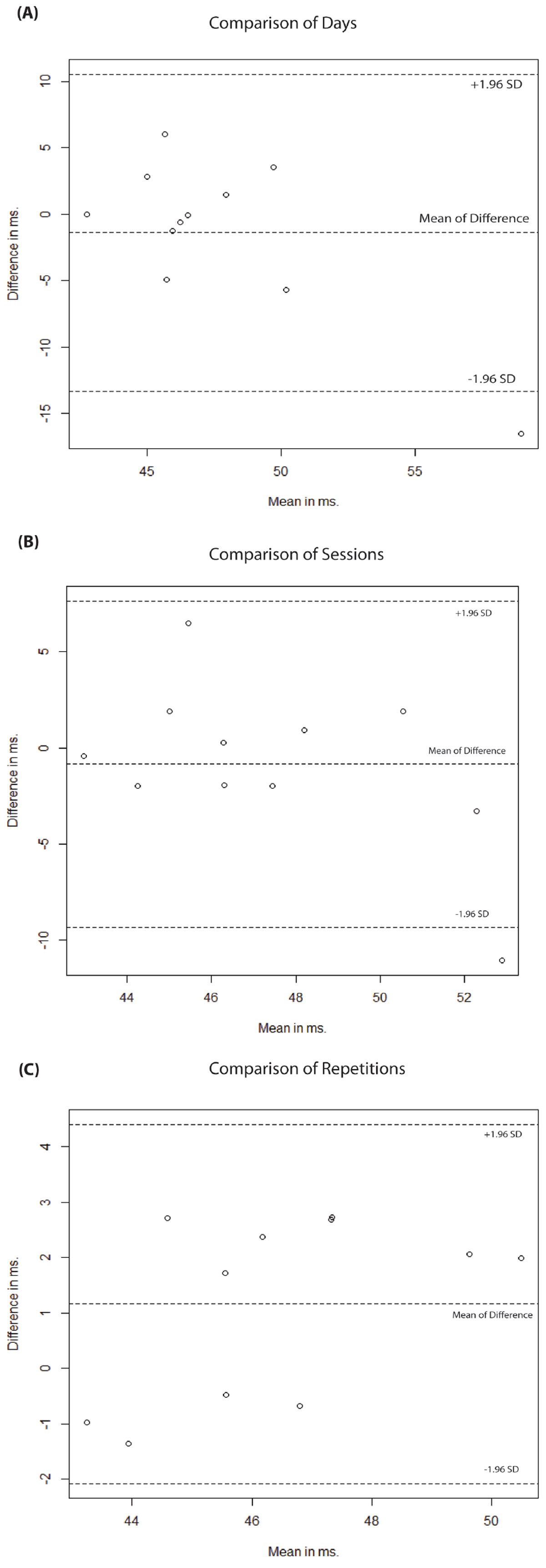
**Bland-Altman plots for comparison of global T2 for first scans per day (A), session (B) and repetition (C)**.

Figure 1 shows mean T_2_ values for different imaging sessions, averaged over all subjects and low CVs *between subjects* (7.2 ± 4.3%). There were low CVs *between days* (6.3 ± 4.0%) and *between sessions* (5.0 ± 4.3%). Fig. 2 shows Bland-Altman plots for T_2_ values between first scan of day 1 and day 2 (A), between first scan of session 1 and session 2 (B), and between scan 1 and 2 within each first session (C).

## Conclusions

Slice-Interleaved T_2_ mapping sequence yields reproducible T_2_ measurements with highest CV of 7.2 ± 4.3% for between day scans.

